# Comparing Clinical Outcomes of COVID-19 and Influenza-Induced Acute Respiratory Distress Syndrome: A Propensity-Matched Analysis

**DOI:** 10.3390/v15040922

**Published:** 2023-04-05

**Authors:** Shiza Virk, Mohammed A. Quazi, Adeel Nasrullah, Aaisha Shah, Evan Kudron, Prabal Chourasia, Anam Javed, Priyanka Jain, Karthik Gangu, Tariq Cheema, Briana DiSilvio, Abu Baker Sheikh

**Affiliations:** 1 Department of Internal Medicine, Allegheny Health Network, Pittsburgh, PA 15212, USA; 2 Department of Mathematics and Statistics, University of New Mexico, Albuquerque, NM 87106, USA; 3 Division of Pulmonology and Critical Care, Allegheny Health Network, Pittsburg, PA 15212, USA; 4 Department of Internal Medicine, University of New Mexico, Albuquerque, NM 87106, USA; 5 Department of Hospital Medicine, Mary Washington Hospital, Fredericksburg, VA 22401, USA; 6 Department of Internal Medicine, University of Kansas Medical Center, Kansas City, KS 66160, USA

**Keywords:** COVID-19, Influenza, ARDS, mechanical ventilation, National Inpatient Sample

## Abstract

Acute respiratory distress syndrome (ARDS) is one the leading causes of mortality and morbidity in patients with COVID-19 and Influenza, with only small number of studies comparing these two viral illnesses in the setting of ARDS. Given the pathogenic differences in the two viruses, this study shows trends in national hospitalization and outcomes associated with COVID-19- and Influenza-related ARDS. To evaluate and compare the risk factors and rates of the adverse clinical outcomes in patients with COVID-19 associated ARDS (C-ARDS) relative to Influenza-related ARDS (I-ARDS), we utilized the National Inpatient Sample (NIS) database 2020. Our sample includes 106,720 patients hospitalized with either C-ARDS or I-ARDS between January and December 2020, of which 103,845 (97.3%) had C-ARDS and 2875 (2.7%) had I-ARDS. Propensity-matched analysis demonstrated a significantly higher in-hospital mortality (aOR 3.2, 95% CI 2.5–4.2, *p* < 0.001), longer mean length of stay (18.7 days vs. 14.5 days, *p* < 0.001), higher likelihood of requiring vasopressors (aOR 1.7, 95% CI 2.5–4.2) and invasive mechanical ventilation (IMV) (aOR 1.6, 95% CI 1.3–2.1) in C-ARDS patients. Our study shows that COVID-19-related ARDS patients had a higher rate of complications, including higher in-hospital mortality and a higher need for vasopressors and invasive mechanical ventilation relative to Influenza-related ARDS; however, it also showed an increased utilization of mechanical circulatory support and non-invasive ventilation in Influenza-related ARDS. It emphasizes the need for early detection and management of COVID-19.

## 1. Introduction

Since its first detection in the United States (US) in January 2020, severe acute respiratory syndrome coronavirus-2 (SARS-CoV-2) has conferred a devastating impact on the general population and on hospital systems. There have been 101 million cases and over one million deaths are a result of COVID-19 infection in the US as of January 2023 [1]. The COVID-19 pandemic was particularly devastating in its first year, 2020. Both SARS-CoV-2 and Influenza viruses can result in potentially life-threatening consequences, leading to hospitalization, critical illness, and death.

Influenza and SARS-CoV-2, while both transmitted primarily through droplets, differ in terms of contagiousness, with SARS-CoV-2 being more contagious. Influenza A viruses primarily infect airway epithelial cells and bind to sialic acid residues, with human strains favoring α-2,6–linked sialic acid. Viral attachment initiates endocytosis and viral RNA release for replication. Influenza infection leads to nonspecific histological changes, inflammation, and proinflammatory cytokine production, with severe cases causing diffuse alveolar damage.

SARS-CoV-2, a member of the Coronaviridae family, binds to the ACE2 receptor on host cells, leading to internalization and subsequent viral replication. The broad tissue distribution of the ACE2 receptor may contribute to the diverse clinical manifestations of COVID-19. Both viruses exhibit different mechanisms of cellular entry and infection, but both ultimately lead to inflammation and damage in the respiratory system.

The incubation period for Influenza is shorter, at a median of 2 days, while SARS-CoV-2 has a median of 5 days. Risk factors for Influenza include age extremes, immunosuppression, pregnancy, and chronic lung and liver diseases. In contrast, SARS-CoV-2 risk factors include advanced age, male sex, obesity, hypertension, and pre-existing conditions such as cardiac diseases, cancers, and type 2 diabetes. Clinical presentations differ, with Influenza commonly causing fever, chills, headache, myalgia, cough, nasal congestion, sore throat, and fatigue. SARS-CoV-2, on the other hand, typically presents with fever, chills, myalgia, shortness of breath, and fatigue. The case fatality rate for Influenza is lower, at 0.1%, while SARS-CoV-2 ranges between 0.25 and 3%. Diagnostically, both infections are detected using nucleic acid amplification tests. Treatment options for Influenza include neuraminidase inhibitors, cap-dependent endonuclease inhibitors, and M2 channel blockers. SARS-CoV-2 treatment varies depending on symptom severity and may involve corticosteroids and immunosuppressants [2].

## 2. Materials and Methods

### 2.1. Data Source

The study was conducted using the US NIS database from 2020. The NIS is the largest all-payer healthcare database in the United States, developed by the Agency of Healthcare Research and Quality Utilization Project [3]. The NIS contains data on approximately 20% of stratified samples of all discharges from US hospitals, which represents more than 97% of the US population, equivalent to 7 to 8 million hospital discharges per annum. This study involved the analysis of deidentified data and, as such, was exempt from institutional review board approval.

### 2.2. Study Population

Briefly, international classification of the diseases tenth revision, clinical modification (ICD-10-CM) diagnosis codes were used to retrieve patient samples with comorbid conditions, and ICD-10 procedure codes were used to identify inpatient procedures (Supplementary Table S1). Our study included all patients who were 18 years of age and older admitted to the hospital with a diagnosis code COVID-19 and Influenza infection (both Influenza A and B) related to ARDS in the study. Exclusion criteria included patients with age less than 18, missing or unavailable data on mortality, and ARDS caused by factors other than COVID-19 and Influenza.

We categorized our patient population based on their exposure status of either COVID-19 or Influenza, and it was then further characterized into three distinct groups of independent variables for adjusted analysis into patient-related variables and hospital-related characteristic variables, and illness-severity-related variables. Patient-related variables included patients’ age, race, sex, comorbidities, insurance status, median income based on patient’s zip code, and disposition; hospital-related variables included location, teaching status, bed size, and region; and illness-severity-related variables included length of stay (LOS), mortality, hospitalization cost, mechanical ventilation, mechanical circulatory support, and vasopressors. The outcomes of the study were also categorized into primary and secondary outcomes.

- The primary outcome was in-hospital mortality.
- Secondary outcomes included:
  1. Invasive and non-invasive mechanical ventilation, i.e., continuous positive pressure ventilation (CPAP) and bilevel positive pressure ventilation (BIPAP);
  2. Complications including vasopressor requirement, sudden cardiac arrest, mechanical circulatory support, venous thromboembolism, cardiogenic shock, hemodialysis need, cardiac arrest, acute liver failure, acute kidney injury, and HD requirement;
  3. Length of stay, mean total hospitalization charge, and patient disposition.

### 2.3. Statistical Analysis

The NIS includes sampling weights from the stratified sampling design that can be used to calculate national estimates and correct variances. Descriptive statistics were used to summarize the continuous and categorical variables. Continuous variables were summarized as mean ± SD (standard deviation), and categorical data as numbers and percentages. Univariate analyses for the significance of between-group comparisons used the Chi-square test for independence for categorical variables (e.g., sex, age group, and risk factors), as shown in Table 1. On the unmatched sample, logistic regression was used to identify independent variables (*p* ≤ 0.2) for binary response variables (e.g., mortality, vasopressor requirement, etc.), and simple linear regression was used for continuous responses (e.g., length of stay and total charges), which were then utilized to build a multivariate regression model. As our case group (C-ARDS) had a significantly higher sample than the control group (I-ARDS), we conducted a secondary analysis on the propensity-matched sample to confirm results obtained by multivariate analysis on the unmatched sample.

In addition, the baseline demographics (age, race, gender, income status, insurance status) and the Elixhauser comorbidities were used as covariates to match samples from control and treatment groups using the propensity scores in R programming language. The 1:1 matching used a generalized linear model (logistic regression) for distance and the greedy nearest neighbor matching method. On the matched cohort (n = 2875), a secondary multivariate regression model was built as described above. SAS was employed in the process of data curation, and the analyses were performed in Python and R.

## 3. Results

**Figure 1:**
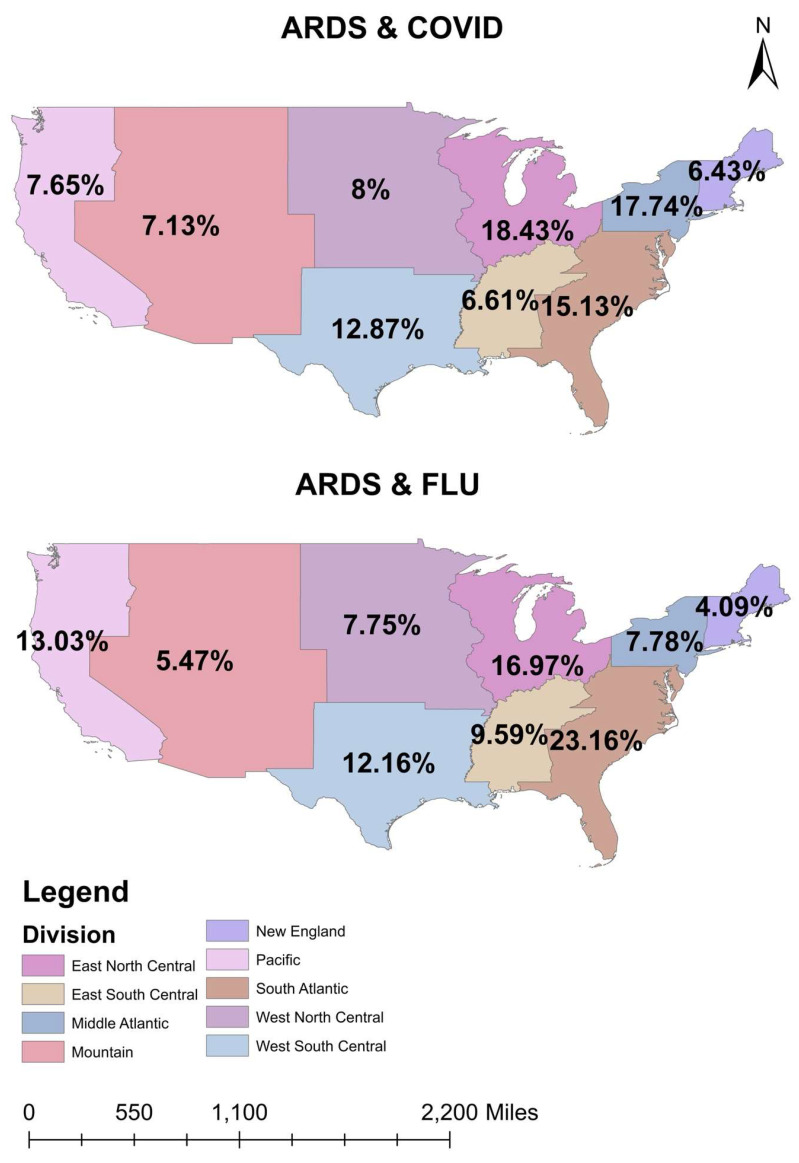
Geographic distribution of ARDS patients with COVID-19 and Influenza in the United States.

Our study population included a total of 106,720 patients with a diagnosis of ARDS during their hospitalization and a diagnosis of COVID-19 or Influenza infection but not both. Of these, 103,845 (97.3%) had COVID-19 and 2875 (2.7%) had Influenza. Patient characteristics, including demographics and comorbidities, are summarized in Table 1. The geographic distribution of ARDS patients with COVID-19 and Influenza infection in the United States are shown in Figure 1.

Males were predominant among patients hospitalized in both C-ARDS (60.7%) and I-ARDS (51.3%). The mean age of patients with C-ARDS was 63–64 years, as compared to 54–58 years in I-ARDS. The 50–69 age group was most affected by both C-ARDS and I-ARDS (46.6% and 47.6%, respectively), followed by the age group of 70 years and above (C-ARDS 37.6% and I-ARDS 21.9%). Significant racial differences were observed between the two cohorts. I-ARDS was disproportionately more prevalent in the white (62.4% vs. 43.6, *p* < 0.001) population. The prevalence of C-ARDS relative to I-ARDS was nearly twice as high in Asian or Pacific Islander (4.4% vs. 2.5%, *p* < 0.001) and Hispanic populations (26.6% vs. 13.6%, *p* < 0.001), and slightly higher in African American (18.3% vs. 17.7%, *p* < 0.001) and Native American populations (2.0% vs. 2.3%, *p* < 0.001). C-ARDS patients were more likely to have Medicare insurance than I-ARDS patients (49.6% vs. 42.0%, *p* < 0.001). C-ARDS was disproportionately more prevalent in patients with diabetes (48.1% vs. 39.5%, *p* < 0.001), obesity (38.1% vs. 28.5%), chronic kidney disease (CKD) (12.8% vs. 8.9%, *p* < 0.004), and dementia (6.7% vs. 3.5%, *p* < 0.001). I-ARDS disproportionately affected patients with a history of malignancy (5.9% vs. 3.8%), drug use (5.7% vs. 1.8%), smoking (35.1% vs. 21.6%), alcohol use disorder (5.4% vs. 2.1%), chronic lung (34.1% vs. 22.3%), and autoimmune disease (5.2% vs. 3.3%), as well as those with Medicaid insurance. Table 1 describes patient characteristics in detail.

All the variables in Table 1 were used to generate a propensity score. We matched 2875 C-ARDS patients with 2875 I-ARDS patients. The matched cohorts were assessed for covariate balance. Propensity matching eliminated almost all the statistically significant differences between the two cohorts in terms of clinical characteristics, demographics, and most of the baseline comorbidities, except for patients with CAD and smoking, who had significantly higher rates of I-ARDS (CAD in 81.7% I-ARDS vs. 12.9% in C-ARDS [*p* = 0.001] and smoking in 35.1% vs. 23.1%, respectively [*p* < 0.001]). Supplementary Table S1 tabulates propensity-matched patient demographics and comorbidities.

### 3.1. In-Hospital Outcomes

A multivariate logistic regression was carried out, and outcomes were adjusted for age, hospital bed size, race, gender, hospital location, hospital teaching status, hospital region, median household income, expected primary payer (insurance status), and Elixhauser comorbidities. This was followed by a propensity score matching (PSM) analysis of all the variables in Table 2.

### 3.2. In-Hospital Mortality

**Figure 2:**
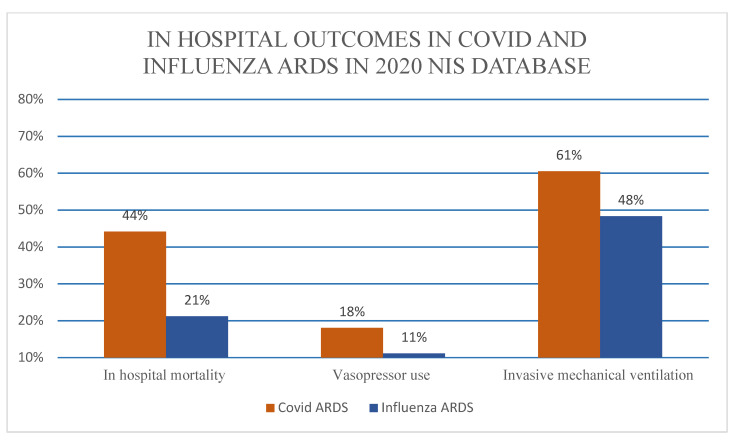
In hospital outcomes in COVID ARDS versus Influenza ARDS.

Patients with C-ARDS had higher in-hospital mortality (50.2% vs. 21.2%, *p* < 0.001) with an adjusted odds ratio (aOR) 4.1 (95% CI 3.3–5.1). Like the unmatched C-ARDS cohort, after PSM C-ARDS patients were noted to have significantly higher in-hospital mortality (44.1% vs. 21.2%, adjusted OR 3.2 (95% CI 2.5–4.2, *p* < 0.001)) (Table 2 and Table 3). Figure 2 illustrates core clinically and statistically significant outcomes.

### 3.3. In-Hospital Complications

Additionally, the C-ARDS cohort had a higher likelihood of requiring vasopressors (17.4% vs. 11.1%, aOR 1.4, 95% CI 1.1–1.9, *p* < 0.001) or invasive mechanical ventilation (59.7% vs. 48.3% aOR 1.5, 95% CI 1.3–1.8, *p* < 0.001). However, the I-ARDS cohort had noticeably higher odds of mechanical circulatory support (7.6% vs. 2.6%, OR 4.7, 95% CI 3.3–6.8, *p* <0.001), although they had nearly 40% less chance of cardiogenic shock. Similarly, C-ARDS patients had a 20% lower chance of being put on a non-invasive mechanical ventilation (OR 0.8, 95% CI 0.4–0.9, *p* <0.023). Table 2 describes key clinical outcomes.

After PSM, similar outcomes were noted with vasopressor use (18.1% vs. 11.1%, aOR 1.7, 95% CI 1.2–2.4, *p* = 0.03) and invasive mechanical ventilation (60.5% vs. 48.3%) in C-ARDS as compared to I-ARDS. However, in patients with C-ARDS, the adjusted odds ratio after propensity match, although statistically significant, indicated a less than 50% chance of mechanical circulatory support in comparison to the I-ARDS cohort (aOR 0.5, 95% CI 0.3–0.98, *p* = 0.043). Similarly, the chances of non-invasive mechanical ventilation dropped from 80% to nearly 70% with aOR 0.7 (95% CI 0.5–0.95) *p* = 0.024). Outcomes can be seen in Table 3.

### 3.4. In-Hospital Quality Measures and Disposition

**Figure 3:**
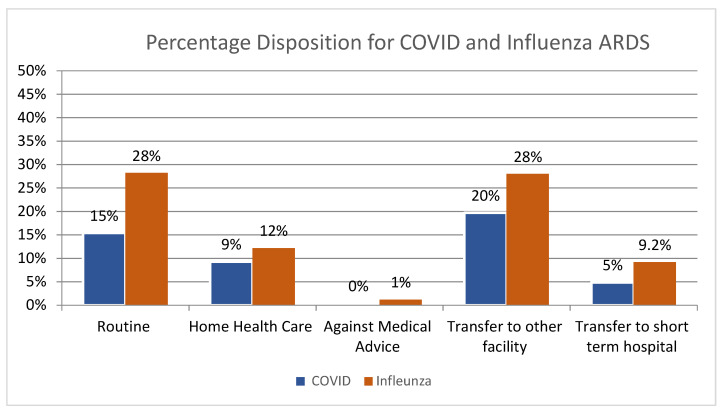
Distribution of disposition of Influenza ARDS in National Inpatient Discharges in 2020.

In the I-ARDS cohort, disposition rates post discharge from hospital were as follows: home (15.3% for C-ARDS vs. 28.1% for I-ARDS, *p* < 0.001), home health care (9.3% C-ARDS vs. 12.2% I-ARDS, *p* < 0.001), LTAC/SNF (19.7% C-ARDS vs. 28% I-ARDS, *p* < 0.001), to short term hospitals (4.9% C-ARDS vs. 9.2% I-ARDS, *p* < 0.001), and against medical advice (0.3% C-ARDS vs. 9.2% I-ARDS, *p* < 0.001), as summarized in Table 2 and Figure 3. However, longer in-hospital lengths of stay were seen with C-ARDS (19.1 days vs. 14.5 days in C-ARDS and I-ARDS, respectively), with a median length of stay of 4.6 days higher.

After PSM, disposition rates remained higher for I-ARDS compared to C-ARDS for discharge home (28.1% vs. 20.5%), home with home health (12.1% vs. 10.1%), against medical advice (1.2% vs. 0.8%), transfer to skilled nursing facility or LTAC (28.0% vs. 16.8%), and transfer to short-term hospital (9.21% vs. 7.47%). A significant difference was found in the total length of stay for C-ARDS, which was nearly 4 days higher (18.8 days vs. 14.5 days, *p* < 0.001). No significant difference was found in mean total hospital charges (*p* = 0.143). Table 3 describes the propensity matched analysis of core outcomes.

### 3.5. Mortality Predictors in COVID-Positive ARDS

**Figure 4:**
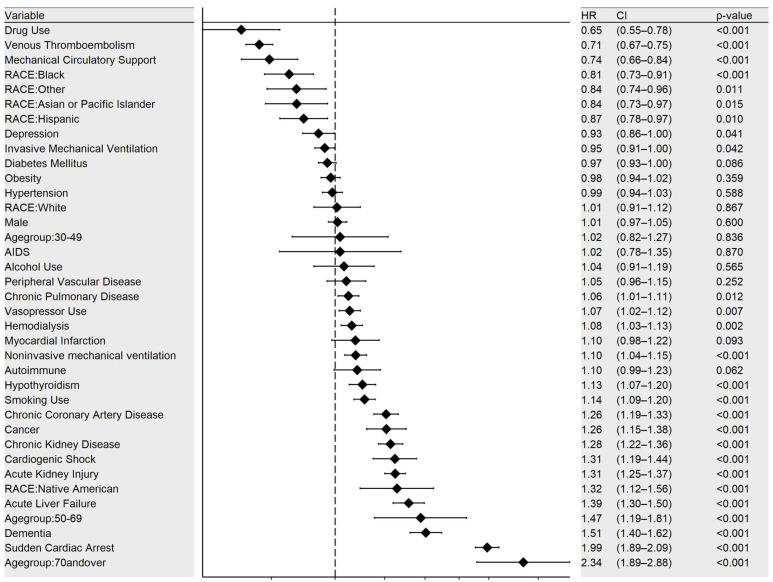
Mortality predictors in COVID-19 patients (multivariate analysis).

Multivariate analysis for predictors of mortality in C-ARDS showed that older patients had higher mortality rates, with HR 1.5 (CI 1.2–1.8, *p* < 0.001) in the 50–69 age group and HR 2.3 (CI 1.9–2.9, *p* < 0.001) in the 70 and above age group. Higher mortality rates were also observed in patients with comorbidities, including chronic pulmonary disease (HR 1.06 [CI 1.02–1.12], *p* = 0.12), hypothyroidism (HR 1.1 [CI 1.1–1.2], *p* < 0.001), smoking (HR 1.1 [1.1–1.2], *p* < 0.001), coronary artery disease (HR 1.3 [1.2–1.3], *p* < 0.001), malignancy (HR 1.3 [CI 1.2–1.4], *p* < 0.001), CKD (HR 1.3 [CI 1.2–1.4], *p* < 0.001), and dementia (HR 1.5 [CI 1.4–1.6], *p* < 0.001). Native Americans had significantly higher mortality rates than any other race, with HR 1.3 [CI 1.2–1.4], *p* < 0.001 (Figure 4).

## 4. Discussion

To our knowledge, this is the most comprehensive study describing clinical characteristics and core outcomes of patients hospitalized with COVID-19-related ARDS and Influenza-related ARDS.

It is important to emphasize that while some outcomes of COVID-19 and Influenza ARDS may be well-known, the comparison between these outcomes on a large scale has not yet been well-studied. As the largest study to date, this research fills a crucial gap in the literature and provides valuable insights into the management and prognosis of ARDS secondary to different viral infections.

The most salient findings of this study include: (1) ARDS patients with COVID-19 had significantly higher in-hospital mortality compared to ARDS with Influenza; (2) patients with COVID-19 and ARDS had a significantly increased need for vasopressors or invasive mechanical ventilation compared to Influenza-related ARDS; (3) patients with Influenza-related ARDS significantly increased the utilization of mechanical circulatory support and non-invasive ventilation as compared to C-ARDS; and (4) predictions of mortality in C-ARDS include the demographic factors old age (>70 years) and Native American race; comorbidities including chronic pulmonary disease, coronary artery disease, CKD, cancer, and dementia; and complications of C-ARDS including liver failure, cardiogenic shock, and acute kidney.

Of 106,720 patients with ARDS and either a COVID-19 or Influenza infection during a hospitalization between 1 January 2020 and December 2020, a large number of patients developed C-ARDS (97%), whereas only 2.7% had I-ARDS, and the net prevalence of C-ARDS was around 6.25%, given the peak of the pandemic as more and more people were becoming infected with COVID-19. A review of the literature published globally in 2020 pooled nearly more than 1000 studies, with total weighted COVID-19 patients of about 2486, suggested that nearly 33% of the patient admitted with COVID-19 developed ARDS [4]. Most likely, the discordance between results may be attributed to the data pool from patients very early in pandemic compared to our study, when disease burden and severity declined with pandemic progression [5]. To date, the world has witnessed frequent epidemics and four major Influenza pandemics during the years 1918, 1957, 1968, and 2009. However, there has been a steady decline in cases of Influenza over past few years through immunizations, effective response teams, global monitoring, mitigation plans, and pandemic interval frameworks proposed by the Center for Disease Control (CDC) [6]. During the COVID-19 pandemic, an unprecedented low incidence of Influenza in the USA and globally were reported by the CDC, with astonishing rates of 1675 (0.2%) positive Influenza tests out of 818,939 specimens collected across the US clinical laboratory compared to previous years (26.2% in 2018, 30.3% in 2019) [7]. One of the proposed explanations for such a decline is largely attributed to COVID-19 mitigation measures, such as wearing face masks, staying home, hand washing, school closures, reduced travel, increased ventilation of indoor spaces, and physical distancing, as vaccination rates were unchanged, and perhaps even declined slightly in some groups [7,8]. This contributed to dramatically fewer Influenza-related illnesses, hospitalizations and deaths compared to the previous Influenza seasons, and also explains the low percentage population affected by I-ARDS compared to COVID-19 ARDS in our study [6,7,9].

Although our study population shows a large difference in the incidences of C-ARDS and I-ARDS, a PSM analysis was performed which still showed nearly three times higher mortality with C-ARDS. This suggests that there must exist some fundamental differences between the two viruses in terms of disease pathogenesis, progression and pre-existing factors explaining the higher mortality in the COVID-19 positive cohort. Multiple retrospective studies, although having smaller sample sizes, have shown similar high mortality in patients with C-ARDS compared to I-ARDS.

A retrospective study of 139 ICU patients with either COVID-19 or Influenza at two Washington state hospitals between 2019 and 2020 demonstrated 40% mortality in COVID-19 ICU patients and 19% in Influenza patients, with an adjusted relative risk of mortality of 2.13 (95% CI 1.24–3.63) [10]. The proposed pathogenetic differences between the two viruses, and therefore the two different types of ARDS, include a greater degree of inflammatory response, increased infectivity, viral mediated dysregulation of the host immune responses, and associated hypercoagulability leading higher rates of life threatening complications [11]. The higher mean age of C-ARDS may also contribute to the higher mortality rate [12]. Routine immunization for Influenza may also provide some degree of protective immunity against Influenza, and hence against I-ARDS; no COVID-19 vaccine was available in the US until December 2020, at which point it was only available to medical professionals [13]. Interestingly, the difference in disease progression can be explained by respiratory physiology in C-ARDS vs. I-ARDS patients, as a German study explains the sharp decline in respiratory system compliance in ventilated C-ARDS patients (40.7 mL/min to 23.87 mL/mbar in 2 weeks, *p* = 0.037) as they are transitioned to venovenous extracorporeal membrane oxygenation (VV-ECMO) compared to I-ARDS [14].

To complicate matters, Influenza and COVID-19 clinically present with overlapping signs and symptoms, making it difficult to distinguish between the two infections; this can therefore lead to delays in diagnosis and disease-specific management [15].

Various comorbidities impact prognosis in COVID-19 patients with advanced age, male sex, and comorbidities such as CKD, all linked to higher mortality [16]. Accordingly, our multi-regression analysis showed demographic factors such as age above 70 years and Native American race and comorbidities including chronic pulmonary disease, CAD, CKD, cancer, and dementia. A riveting finding in our study was that Native American ethnicity was associated with higher mortality in patients with COVID-19. A large cross-sectional study on 18,731 adults admitted to a non-federal, i.e., non IHS (Indian health services) hospital in the state of Mississippi showed, by choosing patients with a comorbidity index of 0, the adjusted odds of in-hospital mortality among African American (aOR, 0.25, 95% CI, 0.18–0.34) and White (aOR, 0.23, 95% CI 0.16–0.31) patients: 75% and 77% less as compared to the Native American population. However, since this study only included patients in a nonfederal hospital, likely a tertiary care center, which tend to have a sicker patient pool, it is a possibility that higher mortality rates are skewed with the given population [17]. Although some Indian Health Service (IHS) facilities offer inpatient care and well-established emergency departments, American Indian and Alaska Native individuals requiring advanced COVID-19 treatment or higher levels of care often had to seek help at non-IHS facilities. The IHS is a federal healthcare program designed to provide tribe members with accessible, cost-free medical care. However, federal reimbursement for care received outside the IHS system necessitates an IHS clinician referral and subsequent validation that the care could not be provided by an IHS facility. The complicated and often lengthy reimbursement process often results in unpaid expenses due to limited funding, leaving an estimated 40% of eligible American Indian and Alaska Native individuals’ healthcare needs uncovered by federal funds [18]. These additional barriers may create double disparity situations for American Indian and Alaska Native residents, such that COVID-19 care was delayed or prevented altogether. Our study highlights this disparity, and there remains a gap in the development of effective frameworks in healthcare to prevent delays in COVID-19-related therapies, and in overall aims to reduce mortality and morbidity in indigenous populations to preserve life along with tribal culture, tradition and language.

Our study shows that the C-ARDS cohort had higher odds for requiring vasopressors and invasive mechanical ventilation compared with I-ARDS patients. Shock in COVID-19 can be a multifactorial entity, which can either be a combination or an isolated manifestation of distributive shock due to bacterial co-infection or the virus itself leading to hyperinflammatory response and loss of vasomotor tone [19]; cardiogenic shock due to either viral mediated myocardial injury, demand ischemia, stress induced cardiomyopathy, or, less commonly, acute plaque rupture [20]; obstructive shock due to VTEs, dynamic hyperventilation related to ARDS, pneumothorax and, rarely, cardiac tamponade [21]; or hypovolemic shock due to poor oral intake, and high grade fever leading to insensible losses [22].

Finally, regional distribution of C-ARDS and I-ARDS correlate to percentages of racial populations in the existing regions and vaccination status of individuals. I-ARDS is more prevalent in the south Atlantic region, where the percentage population of African Americans is high, which co-relates to the lowest rates of Influenza vaccination being in African Americans and Hispanics (38.6–40%) [7,9,23]. Extrapolated results showed, in a retrospective study with 265 patients in ICU, that full vaccination status was associated with lower mortality [61.5% vs. 68.9%, HR 0.55, 5% CI 0.32–0.94, *p* = 0.03] compared with controls, which suggests that vaccination might be beneficial even among patients who were intubated owing to C-ARDS [24]. Similarly, ARDS prevention in Influenza has been historically linked to previous vaccination status [25]. Therefore, the Influenza vaccine is recommended by various national and international health authorities, including the World Health Organization, among high-risk groups, including healthcare workers, to help minimize the Influenza burden as well as allow better preparedness for COVID-19 waves [26]. It is, therefore, essential to highlight the availability and administration of safe and effective vaccines against COVID-19 and Influenza. Crucial measures, such as restoring public trust in vaccines through clear, consistent, transparent, and efficient communication from policymakers, the media, and healthcare professionals, along with engaging communities effectively, are vital for enhancing vaccine acceptance. This highlights the significance of public health campaigns and policy efforts, particularly in the context of large-scale immunization.

Although both COVID-19 and Influenza can lead to ARDS, they induce distinct viral and host immune responses. COVID-19, caused by SARS-CoV-2, is associated with a dysregulated immune response leading to a cytokine storm, while Influenza, caused by the Influenza virus, triggers a different pattern of immune response [27]. This difference in immune activation can impact the severity and progression of ARDS and warrants a direct comparison to better understand the clinical implications. Moreover, the therapeutic choices and outcomes are varied for both entities. The use of corticosteroids has shown benefit in COVID-19 ARDS, reducing mortality and the need for mechanical ventilation [28]. However, corticosteroids have not demonstrated similar benefits in Influenza ARDS and may even be associated with worse outcomes [27]. This comparison is essential for guiding clinicians in making evidence-based therapeutic decisions. Influenza pandemics have shown that different strains can lead to varied outcomes in patients with ARDS. A large-scale study comparing COVID-19 and Influenza ARDS can help determine whether strain-dependent differences also exist in COVID-19, and how these variations impact the clinical course and management of ARDS in both cases. This can be further explored in future studies.

Understanding the differences in outcomes between COVID-19 and Influenza ARDS can provide valuable information for healthcare systems to allocate resources and develop strategies during pandemics or outbreaks. A large-scale comparison between COVID-19 and Influenza ARDS outcomes can lay the groundwork for future research, helping scientists identify potential therapeutic targets, prognostic markers, and prevention strategies.

## 5. Limitations

There are several limitations to our study, including the retrospective nature of the study. First, our data are limited to index hospitalization, and thus information on post-discharge short-term and long-term outcomes is limited. Second, comorbidities and complications were identified using ICD-10 codes that are subject to suboptimal coding or coding errors, which may lead to bias. Third, information about the severity of COVID-19 infection, the variant of COVID-19, and whether a patient is vaccinated was unavailable in this database. Our data are also limited to the year 2020, and since then there have been multiple other COVID variants which may demonstrate a different manifestation of C-ARDS, which might not be as severe as variants such as delta, which predominated in 2020. It is also likely that persistent COVID PCR positivity, a common phenomenon in 2020, was conflated with active COVID infection. In addition, our patient population was limited to US hospitals and thus might not be generalizable worldwide. Given additional data and the constant development of research data regarding COVID-19, coinfection rates, and vaccination data, additional data analysis may be required in the future.

## 6. Conclusions

Based on a nationally representative and large sample of patient data comparing acute respiratory distress syndrome in COVID-19- and Influenza virus-positive cohorts, we found that COVID-19-related ARDS patients have a higher rate of in-hospital deaths and are more likely to require intensive treatments such as vasopressor and ventilatory support compared to those with Influenza-related ARDS. Our study demonstrates the importance of early intervention in ARDS for both COVID-19 and Influenza to prevent inpatient mortality, and aims for early intervention and management.

## Figures and Tables

**Table 1:** COVID-ARDS (C-ARDS) and Influenza-ARDS (I-ARDS): Patient-level characteristics.

Characteristics	C-ARDS, n (%)	I-ARDS, n (%)	*p* Value
n = 106,720	103,845 (97.3)	2875 (2.70)	
Gender (%)			<0.001
Female	40,720 (39.2)	1400 (48.6)	
Male	63,125 (60.7)	1475 (51.3)	
Mean Age Years (SD ^1^)			
Female	64.34 (14.5)	58.66 (17.1)	
Male	63.56 (13.8)	54.91 (14.0)	
AGE Groups Years (%)			<0.001
≥18–29	1850 (1.8)	130 (4.5)	
30–49	14,575 (14.0)	744 (25.9)	
50–69	48,366 (46.5)	1369 (47.6)	
≥70	39,053 (37.6)	630 (21.9)	
RACE (%)			<0.001
White	43,727 (43.6)	1748 (62.4)	
Asian or Pacific Islander	4365 (4.4)	70 (2.5)	
Black	18,339 (18.2)	494 (17.7)	
Hispanic	26,650 (26.5)	378 (13.5)	
Native American	2039 (2.03)	64 (2.3)	
Other	5165 (5.2)	40 (1.4)	
MEDIAN HOUSEHOLD INCOME (%)			0.118
≤USD 49,999	34,801 (34.1)	969 (34.5)	
USD 50,000–64,999	27,869 (27.3)	824 (29.3)	
USD 65,000–85,999	22,870 (22.4)	517 (18.3)	
≥USD 86,000	16,490 (16.1)	500 (17.7)	
INSURANCE STATUS (%)			<0.001
Medicaid	15,565 (15.0)	579 (20.1)	
Medicare	51,376 (49.5)	1211 (42.0)	
No charge	190 (0.18)	25 (8.9)	
Other	4835 (4.7)	76 (2.6)	
Private Insurance	28,205 (27.2)	814 (28.3)	
Self-pay	3444 (3.3)	170 (5.9)	
HOSPITAL DIVISION (%)			<0.001
East North Central	16,095 (15.4)	469 (16.3)	
East South Central	5434 (5.2)	265 (9.2)	
Middle Atlantic	16,677 (16.0)	215 (7.5)	
Mountain	9451 (9.1)	151 (5.2)	
New England	4964 (4.8)	113 (4.0)	
Pacific	12,435 (11.9)	360 (12.5)	
South Atlantic	17,459 (16.8)	640 (22.2)	
West North Central	7984 (7.7)	214 (7.5)	
West South Central	13,346 (12.8)	336 (15.4)	
HOSPITAL BED SIZE (%)			0.510
Large	54,390 (52.4)	1555 (54.0)	
Medium	28,500 (27.4)	795 (27.6)	
Small	20,955 (20.2)	525 (18.2)	
HOSPITAL TEACHING STATUS (%)			0.131
Rural	6330 (6.09)	220 (7.7)	
Urban nonteaching	15,210 (14.6)	470 (16.3)	
Urban teaching	82,305 (79.2)	2185 (76.0)	
COMORBIDITIES (%)			
CAD ^2^	16,770 (83.8)	525 (81.7)	0.175
Myocardial Infarction	3455 (3.3)	110 (3.8)	0.511
Hypertension	69,689 (67.1)	1871 (65.0)	0.298
Diabetes Mellitus	49,944 (48.0)	1131 (39.4)	<0.001
Cancer	4000 (3.9)	170 (5.9)	0.01
Obesity	39,540 (38.0)	820 (28.5)	<0.001
Drug Abuse	1870 (1.8)	165 (5.7)	<0.001
Smoking	22,425 (21.5)	1010 (35.1)	<0.001
Alcohol	2214 (2.1)	156 (5.4)	<0.001
Chronic Pulmonary Disease	23,177 (22.3)	978 (34.0)	<0.001
HIV ^3^	534 (0.5)	16 (0.5)	0.982
Peripheral Vascular Disease	4469 (4.3)	101 (3.5)	0.334
CKD ^4^	13,330 (12.8)	255 (8.9)	0.004
Hypothyroidism	11,760 (11.3)	350 (12.1)	0.526
Autoimmune	3394 (3.3)	151 (5.2)	0.010
Depression	9190 (8.8)	291 (10.0)	0.303
Dementia	7002 (6.7)	102 (3.5)	0.001

^1^ SD = Standard Deviation, ^2^ CAD = coronary artery disease, ^3^ HIV = Human immunodeficiency virus, ^4^ CKD = chronic kidney disease, Autoimmune.

**Table 2:** In-hospital outcomes in COVID-ARDS (C-ARDS) and Influenza-ARDS (I-ARDS).

Variable	C-ARDS (%)	I-ARDS (%)	*p* Value
n = 106,720	103,845 (97.30)	2875 (2.70)	--
In-hospital mortality (N = 52,800)	52,190 (50.25)	610 (21.21)	<0.001
	Adjusted odds ratio ^1^	4.07 (95% CI 3.28–5.09)	
Acute Liver Failure	5770 (5.55)	180 (6.26)	0.429
	Adjusted odds ratio ^1^	0.86 (95% CI 0.62–1.24)	
Sudden Cardiac Arrest	10,221 (9.84)	229 (8.00)	0.258
	Adjusted odds ratio ^1^	1.19 (95% CI 0.88–1.64)	
Vasopressor requirement	18,079 (17.41)	321 (11.13)	0.004
	Adjusted odds ratio ^1^	1.45 (95% CI 1.12–1.92)	
Mechanical Circulatory Support	2696 (2.59)	219 (7.65)	<0.001
	Adjusted odds ratio ^1^	4.68 (95% CI 3.30–6.78)	
Acute Kidney Injury	60,825 (58.57)	1520 (52.86)	0.703
	Adjusted odds ratio ^1^	0.96 (95% CI 0.81–1.15)	
VTE ^2^	12,230 (11.77)	315 (10.95)	0.382
	Adjusted odds ratio ^1^	1.12 (95% CI 0.86–1.49)	
Cardiogenic shock	3013 (2.89)	127 (4.52)	0.023
	Adjusted odds ratio ^1^	0.60 (95% CI 0.41–0.93)	
Hemodialysis	17,630 (16.97)	525 (18.26)	0.119
	Adjusted odds ratio ^1^	0.83 (95% CI 0.67–1.04)	
Invasive Mechanical Ventilation	61,955 (59.66)	1390 (48.34)	<0.001
	Adjusted odds ratio ^1^	1.53 (95% CI 1.29–1.81)	
Non-Invasive Mechanical Ventilation	17,280 (16.64)	545 (18.95)	0.049
	Adjusted odds ratio ^1^	0.80 (95% CI 0.64–1.00)	
Mean total hospitalization charge (USD)	USD 314,910.79	USD 293,528.42	0.203
	Adjusted total charge ^1^	USD 22,379.6 higher	
Mean length of stay (days)	19.11	14.53	<0.001
	Adjusted length of stay ^1^	4.62 days higher	
Disposition			<0.001
Routine	15,974 (15.38)	811 (28.17)	
Home Health Care	9645 (9.29)	350 (12.17)	
Against Medical Advice	383 (0.37)	37 (1.21)	
Transfer other (SNF/LTAC)	20,474 (19.71)	801 (28.00)	
Transfer to short-term hospital	5090 (4.90)	265 (9.21)	
Died in Hospital	52,190 (50.27)	610 (21.21)	
Discharged alive unknown destination	55 (0.05)	0 (0)	

^1^ Adjusted for age, hospital bed size, race, gender, hospital location, hospital teaching status, hospital region, median household income, expected primary payer (insurance status), Elixhauser comorbidities. ^2^ VTE = Venous thromboembolism.

**Table 3:** COVID 19 ARDS (C-ARDS) and Influenza ARDS (I-ARDS): propensity 1:1 matched in hospital outcomes.

Variable	C-ARDS (%)	I-ARDS (%)	*p* Value
n = 5750	2875	2875	--
In-hospital mortality (N = 1880)	1270 (44.17)	610 (21.21)	<0.001
	Adjusted odds ratio ^1^	3.21 (95% CI 2.46–4.22)	
Acute Liver Failure	215 (7.47)	180 (6.26)	0.449
	Adjusted odds ratio ^1^	1.19 (95% CI 0.75–1.91)	
Sudden Cardiac Arrest	285 (9.91)	229 (8.00)	0.254
	Adjusted odds ratio ^1^	1.27 (95% CI 0.84–1.93)	
Vasopressor requirement	520 (18.08)	321 (11.13)	0.003
	Adjusted odds ratio ^1^	1.68 (95% CI 1.18–2.41)	
Mechanical Circulatory Support	115 (4.00)	219 (7.65)	0.043
	Adjusted odds ratio ^1^	0.54 (95% CI 0.30–0.98)	
Acute Kidney Injury	1525 (53.04)	1520 (52.86)	0.955
	Adjusted odds ratio ^1^	0.99 (95% CI 0.77–1.27)	
Venous Thromboemolism	330 (11.47)	315 (10.95)	0.770
	Adjusted odds ratio ^1^	1.05 (95% CI 0.72–1.54)	
Cardiogenic shock	105 (3.65)	127 (4.52)	0.516
	Adjusted odds ratio ^1^	0.81 (95% CI 0.43–1.51)	
Hemodialysis	445 (15.47)	525 (18.26)	0.148
	Adjusted odds ratio ^1^	0.78 (95% CI 0.56–1.08)	
Invasive Mechanical Ventilation	1740 (60.52)	1390 (48.34)	<0.001
	Adjusted odds ratio ^1^	1.63 (95% CI 1.27–2.08)	
Non-Invasive Mechanical Ventilation	385 (13.39)	545 (18.95)	0.024
	Adjusted odds ratio ^1^	0.68 (95% CI 0.49–0.95)	
Mean total hospitalization charge (USD)	USD 331,233.49 Adjusted total charge ^1^	USD 293,528.42 = USD 46,097 higher	0.143
Mean length of stay (days)	18.79 Adjusted length of stay ^1^	14.53 = 3.94 days higher	<0.001
Disposition			<0.001
Routine	590 (20.52)	811 (28.17)	
Home Health Care	290 (10.08)	350 (12.17)	
Against Medical Advice	25 (0.86)	37 (1.21)	
Transfer other	485 (16.86)	801 (28.00)	
Transfer to short-term hospital	215 (7.47)	265 (9.21)	

^1^ Adjusted for age, hospital bed size, race, gender, hospital location, hospital teaching status, hospital region, median household income, expected primary payer (insurance status), Elixhauser comorbidities.

## Data Availability

Not applicable.

## Supplementary Materials

The following supporting information can be downloaded at: https://www.mdpi.com/article/10.3390/v15040922/s1, Table S1: ICD 10 codes; Table S2: Propensity 1:1 Matched Patient Characteristics

## References

[1] CDC COVID Data Tracker. https://covid.cdc.gov/covid-data-tracker/#trends_totalcases_select_00.

[2] Solomon D.A., Sherman A.C., Kanjilal S. (2020). Influenza in the COVID-19 Era. JAMA.

[3] NIS Database Documentation. https://hcup-us.ahrq.gov/db/nation/nis/nisdbdocumentation.jsp.

[4] Tzotzos S.J., Fischer B., Fischer H., Zeitlinger M. (2020). Incidence of ARDS and outcomes in hospitalized patients with COVID-19: A global literature survey. Crit. Care.

[5] Christie A., Henley S.J., Mattocks L., Fernando R., Lansky A., Ahmad F.B., Adjemian J., Anderson R.N., Binder A.M., Carey K. (2021). Decreases in COVID-19 Cases, Emergency Department Visits, Hospital Admissions, and Deaths Among Older Adults Following the Introduction of COVID-19 Vaccine—United States, September 6, 2020–May 1, 2021. Morb. Mortal. Wkly. Rep..

[6] Pandemic Influenza. Pandemic Influenza (Flu) CDC. https://www.cdc.gov/flu/pandemic-resources/index.htm.

[7] CDC 2020–2021 Flu Season Summary. https://www.cdc.gov/flu/season/faq-flu-season-2020-2021.htm.

[8] Rubin R. (2021). Influenza’s Unprecedented Low Profile During COVID-19 Pandemic Leaves Experts Wondering What This Flu Season Has in Store. JAMA.

[9] Flu Vaccination Coverage, United States, 2020–2021 Influenza Season. https://www.cdc.gov/flu/fluvaxview/coverage-2021estimates.htm.

[10] Cobb N.L., Sathe N.A., Duan K.I., Seitz K.P., Thau M.R., Sung C.C., Morrell E.D., Mikacenic C., Kim H.N., Liles W.C. (2021). Comparison of Clinical Features and Outcomes in Critically Ill Patients Hospitalized with COVID-19 versus Influenza. Ann. Am. Thorac. Soc..

[11] Torres Acosta M.A., Singer B.D. (2020). Pathogenesis of COVID-19-induced ARDS: Implications for an ageing population. Eur. Respir. J..

[12] Chiumello D., Modafferi L., Fratti I. (2022). Risk Factors and Mortality in Elderly ARDS COVID-19 Compared to Patients without COVID-19. J. Clin. Med..

[13] Valkenburg S.A., Poon L.L.M. (2022). Exploring the landscape of immune responses to influenza infection and vaccination. Nat. Med..

[14] Kronibus N., Seiler F., Danziger G., Muellenbach R.M., Reyher C., Becker A.P., Kamphorst M., Rixecker T.M., Metz C., Bals R. (2022). Respiratory Physiology of COVID-19 and Influenza Associated Acute Respiratory Distress Syndrome. J. Clin. Med..

[15] Bray L., Meznikova K., James D., Mason P., Rislan R., Shah R., Staniland T., Lillie P., Barlow G., Easom N. (2022). 36 Misdiagnoses in the context of suspected pandemic influenza or COVID-19: A systematic review. Clin. Infect. Pract..

[16] Shi C., Wang L., Ye J., Gu Z., Wang S., Xia J., Xie Y., Li Q., Xu R., Lin N. (2021). Predictors of mortality in patients with coronavirus disease 2019: A systematic review and meta-analysis. BMC Infect. Dis..

[17] Musshafen L.A., El-Sadek L., Lirette S.T., Summers R.L., Compretta C., Dobbs T.E. (2022). In-Hospital Mortality Disparities Among American Indian and Alaska Native, Black, and White Patients With COVID-19. JAMA Netw. Open.

[18] American Indians and Alaska Natives Are Dying of COVID-19 at Shocking Rates. https://www.brookings.edu/research/american-indians-and-alaska-natives-are-dying-of-covid-19-at-shocking-rates/.

[19] Bhimraj A., Morgan R.L., Shumaker A.H., Lavergne V., Baden L., Cheng V.C.-C., Edwards K.M., Gandhi R., Muller W.J., O’Horo J.C. (2020). Infectious Diseases Society of America Guidelines on the Treatment and Management of Patients with COVID-19. Clin. Infect. Dis..

[20] Inciardi R.M., Lupi L., Zaccone G., Italia L., Raffo M., Tomasoni D., Cani D.S., Cerini M., Farina D., Gavazzi E. (2020). Cardiac Involvement in a Patient With Coronavirus Disease 2019 (COVID-19). JAMA Cardiol..

[21] Llitjos J.-F., Leclerc M., Chochois C., Monsallier J.-M., Ramakers M., Auvray M., Merouani K. (2020). High incidence of venous thromboembolic events in anticoagulated severe COVID-19 patients. J. Thromb. Haemost..

[22] Silversides J.A., Major E., Ferguson A.J., Mann E.E., McAuley D.F., Marshall J.C., Blackwood B., Fan E. (2017). Conservative fluid management or deresuscitation for patients with sepsis or acute respiratory distress syndrome following the resuscitation phase of critical illness: A systematic review and meta-analysis. Intensive Care Med..

[23] Grohskopf L.A., Blanton L.H., Ferdinands J.M., Chung J.R., Broder K.R., Talbot H.K., Morgan R.L., Fry A.M. (2022). Prevention and Control of Seasonal Influenza with Vaccines: Recommendations of the Advisory Committee on Immunization Practices—United States, 2022–2023 Influenza Season. MMWR Recomm. Rep..

[24] Grapsa E., Adamos G., Andrianopoulos I., Tsolaki V., Giannakoulis V.G., Karavidas N., Giannopoulou V., Sarri K., Mizi E., Gavrielatou E. (2022). Association Between Vaccination Status and Mortality Among Intubated Patients With COVID-19-Related Acute Respiratory Distress Syndrome. JAMA Netw. Open.

[25] Chow E.J., Doyle J.D., Uyeki T.M. (2019). Influenza virus-related critical illness: Prevention, diagnosis, treatment. Crit. Care.

[26] CDC Routine and Influenza Immunization Services During the COVID-19 Pandemic: Interim Guidance. https://www.cdc.gov/vaccines/pandemic-guidance/index.html#:~:text=For%20the%202021%E2%80%932022%20influenza,during%20the%20COVID%2D19%20pandemic.

[27] Zhou Y., Fu X., Liu X., Huang C., Tian G., Ding C., Wu J., Lan L., Yang S. (2020). Use of corticosteroids in influenza-associated acute respiratory distress syndrome and severe pneumonia: A systemic review and meta-analysis. Sci. Rep..

[28] Horby P., Lim W.S., Emberson J.R., Mafham M., Bell J.L., Linsell L., Staplin N., Brightling C., Ustianowski A., Recovery Collaborative Group (2021). Dexamethasone In Hospitalized Patients With Covid-19. N. Engl. J. Med..

