# Supplementary material for: Comparing Clinical Outcomes of COVID-19 and Influenza-Induced Acute Respiratory Distress Syndrome: A Propensity-Matched Analysis
Source: Viruses. 2023 Apr 5;15(4):922. doi: 10.3390/v15040922 (PMC10144713; doi:10.3390/v15040922)
Supplement: Supplementary file 1 [file viruses-15-00922-s001.zip › viruses-2291861-supplementary.pdf]

Supplementary Table S1: ICD 10 Codes

| Disease/Procedure | ICD Codes |
|---|---|
| Acute respiratory distress syndrome | J810, J80 |
| COVID-19 | U071, U00, U49, U50, U85, J1282 |
| Influenza | J09X1, J09X2, J09X3, J09X9, |
| | J1000, J101,J1008,J101,J102,J1081, |
| | J1082,J1083,J1089,J1100,J1108,J111, |
| | J112,J1181,J1182,J1183,J1189 |
| Vasopressor | 3E030XZ,3E033XZ,3E040XZ,3E043XZ,3E050XZ,3E053XZ,3E060XZ,3E063XZ |
| Mechanical Circulatory Support | 5A02110, 5A02210, 5A0211D, 02HA3RZ, 5A02116, 5A0221D, |
| | 5A1522F, 5A1522G, 5A1522H, 5A15A2F, 5A15A2G, 5A15A2H, 5A15223 |
| Mechanical ventilation invasive | 0BH17EZ, 0BH18EZ, 5A1935Z, 5A0945Z, 5A0955Z |
| Non-invasive mechanical ventilation | 5A09457, 5A09458, 5A09358, 5A09557, 5A09558, 5A09357 |
| Sudden cardiac arrest | I462, I468, I469 |
| hemodialysis | 5A1D70Z,5A1D90Z,5A1D80Z,5A1D00Z,5A1D60Z |
| Cardiogenic Shock | R570 |
| Chronic Kidney Disease | N181, N182, N1830, N1831, N1832, N184, N185, N189 |
| Acute kidney injury | N170,N171,N172,N178,N179, N990 |
| Acute liver failure | K7200, K7201, K712 |
| Venous thromboembolism | I82210,I82220,I82290,I82401,I82402,I82403,I82409,I82411, |
| | I82412,I82413,I82419,I82421,I82422,I82423,I82429,I82431,I82432, |
| | I82433,I82439,I82441,I82442,I82443,I82449,I82451,I82452, |
| | I82453,I82459,I82461,I82462,I82463,I82469,I82491,I82492,I82493, |
| | I82499,I824Y1,I824Y2,I824Y3,I824Y9,I824Z1,I824Z2,I824Z3,I824Z9, |
| | I82601,I82602,I82603,I82609,I82611,I82612,I82613,I82619,I82621, |
| | I82622,I82623,I82629,I82890,I8290,I82A11,I82A12,I82A13,I82A19, |
| | I82B11,I82B12,I82B13,I82B19,I82C11,I82C12,I82C13,I82C19,I1260, |
| | I2601,I2602,I2690,I2692,I2693,I2694,I2699,I2609 |
| Smoking | F17, F172, F1720, F17200, F17201, F17203, F17208, |

| | |
|---|---|
| | F17209, F1721, F17210, F17211, F17213, F17218, |
| | F17219, F1722, F17220, F17221, F17223, F17228, |
| | F17229, F1729, F17290, F17291, F17293, F17298, F17299, Z87891 |
| History of percutaneous coronary intervention | Z9861, Z9861 |
| History of Coronary Artery Bypass Surgery | Z951 |
| Previous Myocardial Infarction | I252 |
| Coronary Artery Disease | I2510, I25111, I25118, I25119, I252, I253, I254, |
| | I2541, I2542, I255, I256, I257, I2570, I25700, |
| | I25701, I25708, I25709, I2571, I25710, I25711, I25718, |
| | I25719, I2572, I25720, I25721, I25728, I25729, I2573, I25730, |
| | I25731, I25738, I25739, I2575, I25750, I25751, I25758, I25759, |
| | I2576, I25760, I25761, I25768, I25769 |
| Chronic Pulmonary Disease | Elixhauser comorbidities index |
| Diabetes | Elixhauser comorbidities index |
| AIDS | Elixhauser comorbidities index |
| | |
| Hypothyroidism | Elixhauser comorbidities index |
| Autoimmune | Elixhauser comorbidities index |
| Dementia | Elixhauser comorbidities index |
| Depression | Elixhauser comorbidities index |
| | |
| Lymphoma | Elixhauser comorbidities index |
| Leukemia | Elixhauser comorbidities index |
| Metastatic Cancer | Elixhauser comorbidities index |
| Solid Tumor Without Metastasis (2 kinds) | Elixhauser comorbidities index |
| | |
| Obesity | Elixhauser comorbidities index |
| Drug Abuse | Elixhauser comorbidities index |
| Hypertension | Elixhauser comorbidities index |

| | |
|---|---|
| PAD Peripheral vascular disease | Elixhauser comorbidities index |
| Alcohol | Elixhauser comorbidities index |

**Supplementary Table S2: COVID 19 ARDS (C-ARDS) and Influenza ARDS (I-ARDS): propensity 1:1 matched patient level characteristic. SD=Standard Deviation.**

| Characteristics | C-ARDS (%) | I-ARDS (%) | p value |
|---|---|---|---|
| n = 5,750 | 2,875 | 2,875 | -- |
| Gender (%) | | | 0.555 |
| Female | 1,450 (50.43) | 1,400 (48.69) | |
| Male | 1,425 (49.56) | 1,475 (51.30) | |
| Mean Age Years (SD) | | | -- |
| Female | 56.45 (17.11) | 58.66 (17.15) | |
| Male | 57.37 (14.11) | 54.91 (14.06) | |
| AGE Groups (%) | | | 0.455 |
| >=18-29 | 175 (6.08) | 130 (4.50) | |
| 30-49 | 660 (22.95) | 744 (25.91) | |
| 50-69 | 1,425 (49.56) | 1,369 (47.65) | |
| >=70 | 615 (21.39) | 630 (21.91) | |
| RACE (%) | | | 0.951 |
| White | 1,770 (62.54) | 1,748 (62.43) | |
| Asian or Pacific Islander | 65 (2.29) | 70 (2.50) | |
| Black | 475 (16.78) | 494 (17.71) | |
| Hispanic | 420 (14.84) | 378 (13.59) | |
| Native American | 50 (1.76) | 64 (2.32) | |
| Other | 50 (1.76) | 40 (1.43) | |

| | | | |
|---|---|---|---|
| MEDIAN HOUSEHOLD INCOME (%) | | | 0.976 |
| ≤$49,999 | 1,000 (35.52) | 969 (34.51) | |
| $50,000-64,999 | 800 (28.41) | 824 (29.35) | |
| $65,000-85,999 | 505 (17.93) | 517 (18.32) | |
| ≥$86,000 | 510 (18.11) | 500 (17.79) | |
| INSURANCE STATUS (%) | | | 0.990 |
| Medicaid | 560 (19.47) | 579 (20.17) | |
| Medicare | 1,235 (42.95) | 1,211 (42.08) | |
| No charge | 20 (0.69) | 25 (8.86) | |
| Other | 80 (2.78) | 76 (2.60) | |
| Private Insurance | 830 (28.86) | 814 (28.34) | |
| Self-pay | 150 (5.21) | 170 (5.91) | |
| HOSPITAL DIVISION (%) | | | <0.001 |
| East North Central | 530 (18.43) | 469 (16.34) | |
| East South Central | 190 (6.60) | 265 (9.21) | |
| Middle Atlantic | 510 (17.73) | 215 (7.47) | |
| Mountain | 205 (7.13) | 151 (5.21) | |
| New England | 185 (6.43) | 113 (4.00) | |
| Pacific | 220 (7.65) | 360 (12.52) | |
| South Atlantic | 435 (15.13) | 640 (22.26) | |
| West North Central | 230 (8.00) | 214 (7.47) | |
| West South Central | 370 (12.86) | 336 (15.47) | |
| HOSPITAL BED SIZE (%) | | | 0.055 |

| | | | |
|---|---|---|---|
| Large | 1,525 (53.04) | 1,555 (54.08) | |
| Medium | 675 (23.47) | 795 (27.65) | |
| Small | 675 (23.47) | 525 (18.26) | |
| HOSPITAL TEACHING STATUS (%) | | | 0.331 |
| Rural | 250 (8.69) | 220 (7.65) | |
| Urban nonteaching | 385 (13.39) | 470 (16.34) | |
| Urban teaching | 2,240 (77.91) | 2,185 (76.00) | |
| COMORBIDITIES (%) | | | |
| Coronary Artery Disease | 335 (11.65) | 525 (81.73) | 0.001 |
| Myocardial infarction | 85 (2.95) | 110 (3.82) | 0.415 |
| Hypertension | 1,880 (65.39) | 1,871 (65.04) | 0.901 |
| Diabetes | 1,100 (38.26) | 1,131 (39.47) | 0.671 |
| Cancer | 175 (6.08) | 170 (5.91) | 0.901 |
| Obesity | 750 (26.08) | 820 (28.52) | 0.354 |
| Drug Abuse | 140 (4.86) | 165 (5.73) | 0.510 |
| Smoking | 665 (23.13) | 1,010 (35.13) | <0.001 |
| Alcohol | 125 (4.34) | 156 (5.39) | 0.411 |
| Chronic Pulmonary Disease | 980 (34.08) | 980 (34.08) | 1.000 |
| HIV | 0 (0.00) | 16 (0.52) | 0.082 |
| Peripheral Vascular Disease | 95 (3.30) | 101 (3.47) | 0.870 |
| CKD | 305 (10.60) | 255 (8.87) | 0.319 |
| Hypothyroidism | 335 (11.65) | 350 (12.17) | 0.784 |
| Autoimmune | 135 (4.69) | 151 (5.21) | 0.683 |

| | | | |
|---|---|---|---|
| Depression | 280 (9.73) | 291 (10.08) | 0.843 |
| Dementia | 70 (2.43) | 102 (3.47) | 0.297 |
